# Thymoquinone (TQ) Inhibits Inflammation and Migration of THP-1 Macrophages: Mechanistic Insights into the Prevention of Atherosclerosis Using In-Vitro and In-Silico Analysis

**DOI:** 10.3390/cimb44040120

**Published:** 2022-04-15

**Authors:** Etimad Huwait, Nouf Al-Gharawi, Maryam A. Al-Ghamdi, Mamdooh Gari, Alexandre Prola, Peter Natesan Pushparaj, Gauthaman Kalamegam

**Affiliations:** 1 Department of Biochemistry, Faculty of Science, King Abdul Aziz University, Jeddah 21589, Saudi Arabia; noufalgharawi@hotmail.com (N.A.-G.); maaalghamdi3@kau.edu.sa (M.A.A.-G.); 2 Cell Culture Lab, Experimental Biochemistry Unit, King Fahad Medical Research Centre, King Abdul Aziz University, Jeddah 22252, Saudi Arabia; 3 Center of Excellence in Genomic Medicine Research, King Abdulaziz University, Jeddah 21589, Saudi Arabia; mgari@kau.edu.sa (M.G.); pnatesan@kau.edu.sa (P.N.P.); 4 Department of Medical Laboratory Technology, Faculty of Applied Medical Sciences, King Abdulaziz University, Jeddah 21589, Saudi Arabia; 5 Department of Cell Physiology and Metabolism, Faculty of Medicine, University of Geneva, 1 rue Michel-Servet, CH-1211 Geneva, Switzerland; alexandre.prola@gmail.com; 6 Department of Pharmacology, Saveetha Dental College and Hospital, Saveetha Institute of Medical and Technical Scinences, Chennai 600077, India; 7 Pharmaceutical Division, Nibblen Life Sciences Private Limited, Chennai 600061, India; 8 RMD Specialties Hospital, RMD Academy for Health (A Unit of Pain and Palliative Care Trust), Chennai 600017, India

**Keywords:** thymoquinone, atherosclerosis, THP-1 macrophages, IFN-γ, monocytes migration, cholesterol efflux

## Abstract

Atherosclerosis is an inflammatory disease mediated by interferon (IFN-γ) in concert with cell adhesion molecules and chemokines. Thymoquinone (TQ), a flavonoid derived from *Nigella sativa*, is reported to have anti-inflammatory, antioxidant, and cardiovascular protective properties. We evaluated the effects of TQ on the key pathogenic stages of atherosclerosis, including cell viability, inflammatory gene expression, cell migration, and cholesterol efflux, on human THP-1 macrophages in-vitro. Moreover, in-silico analysis was performed to predict the molecular targets and signaling mechanisms. We demonstrated that TQ treatment had no effect on cell viability and decreased the expression of monocyte chemoattractant protein (MCP-1) and intercellular adhesion molecule (ICAM-1) in response to IFN-γ. In addition, we have also demonstrated that the THP-1 cell migration was inhibited by TQ in the absence or presence of MCP-1. Thymoquinone had no effect on cholesterol efflux from monocytes. In-silico analysis also identified several putative targets for TQ that are associated with inflammatory diseases and associated signaling pathways. Collectively, these results suggest that TQ has anti-inflammatory effects and may be a potential nutraceutical candidate for the prevention and treatment of atherosclerosis.

## 1. Introduction

Atherosclerosis (AS) is a lipid-driven disease characterized by chronic inflammation within the vascular walls of large and medium arteries [1]. It is the most common type of coronary artery disease and a leading cause of morbidity and mortality worldwide [2]. During disease progression, an atherosclerotic plaque is formed as a result of lesions developing in the vascular wall [3]. Atherosclerotic plaque rupture causes thrombosis, which is one of the most common causes of mortality worldwide. The complications of thrombosis include stroke, gangrene, and angina/myocardial infarction. One of the important factors in controlling the incidence of plaque rupture is plaque stability [1]. Atherosclerosis is a chronic inflammation within the vascular wall that is associated with the release of pro- or anti-atherogenic cytokines at different stages of the disease [4,5]. High levels of pro-atherogenic/pro-inflammatory cytokines, such as tumor necrosis factor-alpha (TNF-α), interferon-gamma (IFN-γ), and interleukin-1 (IL-1), are implicated in atherosclerotic lesions. T-lymphocytes and macrophages are the major sources of cytokine production in atherosclerotic lesions [4,5].

An early step in atherosclerosis is the transformation of macrophages into foam cells through the uptake of cholesterol [4,5]. The main initiating step in this disease is the activation of endothelial cells (ECs). The major cause of EC activation is the accumulation of low-density lipoprotein (LDL) in the subendothelial matrix. The plasma concentration of LDL is correlated with the frequency of EC activation [5]. Upon activation, the ECs then express many chemoattractant molecules [3], and the monocytes from the bloodstream are attracted to the activation site, leading to the formation of fatty streaks [6]. On the surface of ECs, monocytes are initially attracted by adhesion molecules, such as P- and E-selectin, expressed on the luminal surface of the activated endothelium and interact with L-selectin molecules on the surface of monocytes [7,8]. The interaction between adhesion molecules and selectins causes monocytes to roll across the endothelial surface. Vascular inflammation stimulates the expression of adhesion molecules, such as intercellular adhesion molecule 1 (ICAM-1) and vascular cell adhesion molecule 1 (VCAM-1) in ECs, which initiate binding with integrins, causing a higher level of adhesion to the endothelium. Migration through the endothelial layer to the intima is then initiated and aided by ECs that express chemokines, such as monocyte chemoattractant protein 1 (MCP-1), TNF-α, and TGF-β [7,8].

Nutraceuticals represent a promising therapeutic target for the prevention of atherosclerosis. In folk medicine, *Nigella sativa* (*NS*) seeds, which are widely known herbal medicines, have been used mainly against obesity and hypertension. The main bioactive constituent of the volatile oil of *NS* seeds is thymoquinone (TQ) (2-isopropyl-5-methyl-1, 4-benzoquinone) [9]. TQ is a flavonoid reported to have anti-inflammatory, anti-oxidant, and lipid metabolic functions [10]. TQ attenuates the development of atherosclerosis in cholesterol-fed rabbits, and this was associated with reduced serum levels of triacylglycerols and LDL. In addition, there has been an increase in high-density lipoprotein (HDL) levels and glutathione content [10,11,12]. TQ also improves aging-related endothelial dysfunction in the rat mesenteric artery [13]. However, the anti-atherogenic effects of TQ and the molecular mechanisms underlying these effects are not fully understood. A better characterization of the effect of TQ is thus necessary for the development of nutraceutical-based approaches for the prevention and treatment of atherosclerosis.

In the present study, we aimed to determine the molecular mechanisms of TQ against atherosclerosis in-vitro in human monocytes and macrophages (THP-1 cell line). We assessed the effects of TQ on cell viability, IFNγ-induced expression of MCP-1 and ICAM-1 genes, monocyte migration, cholesterol efflux, and the molecular targets and signaling pathways using in-silico analysis.

## 2. Materials and Methods

TQ (purity, 98% HPLC), Phorbol 12-myristate13-acetate (PMA), dimethyl sulfoxide (DMSO), and other chemicals were purchased from Sigma Aldrich Chemical Co (Gillingham, UK). All cell culture reagents, media, and supplements were purchased from Gibco (Grand Island, NY, USA). The THP-1 cell line was purchased from the European Collection of Authenticated Cell Cultures (ECACC, Salisbury, UK). Cell culture flasks, multi-well plates, and other tissue-culture plastics were purchased from SPL. The cholesterol efflux assay kit (ab196985) was purchased from (Abcam, Cambridge, UK). The LDH cytotoxicity assay kit (Cat No. 88954) was acquired from (Thermo Fisher Scientific, Wilmington, DE, USA). The RNeasy Mini Kit (Cat No. 74104) and QuantiFast SYBR Green PCR Kit (Cat No. 204054) were obtained from (Qiagen, Manchester, UK). The ImProm-II Reverse Transcription System (Cat No. A3800) was obtained from (Promega, Madison, WI, USA).

### 2.1. Culture and Maintenance of THP-1 Cells

THP-1 cells were cultured in RPMI-1640 medium (Cat. No. A1049101) supplemented with 100 U/mL penicillin, 100 mg/mL streptomycin (Cat No. 15140122), and 10% (*v/v*) heat-inactivated fetal bovine serum (FBS, Cat No. A3160802). The cells were maintained at 37 °C in a humidified atmosphere containing 5% CO_2_. Cell counts and viability were estimated using trypan blue vital stain (0.4%; Cat No. 15250-061, GIBCO, Grand Island, NY, USA). THP-1 cells were maintained in a logarithmic growth phase at a concentration between 105 and 106 cells/mL with periodic medium addition/changes every 2–4 days.

### 2.2. Differentiation of THP-1 Monocytes into Macrophages

THP-1 cells in the exponential growth phase with a viability of 95% were used for the viability assays. Cells were seeded at a density of 4 × 10^4^ cells/100 μL/well in 96-well plates. THP-1 monocytes were differentiated into macrophages using 0.16 µM of phorbol 12-myristate 13-acetate (PMA) for 24 h to enable differentiation into macrophages. THP-1-derived macrophages were treated with various concentrations of TQ (2.5, 5, 7.5, and 10 µM) in 100 µL of culture medium. The cells were incubated for 24 h in a humidified 5% CO_2_ incubator at 37 °C. The TQ working concentrations were prepared by dilution of the stock solutions in culture medium and filter-sterilization with 0.22 μm Millex-GP syringe filters. The corresponding DMSO concentrations were similarly prepared as the vehicle controls.

### 2.3. Lactate Dehydrogenase (LDH) Assay

The lactate dehydrogenase (LDH) in vitro assay kit was used to evaluate cytotoxicity, following the manufacturer’s instructions. The assay measures membrane integrity as a function of the amount of cytoplasmic LDH released into the medium. THP-1 cells were seeded and treated with TQ as briefed earlier for 24 h. The cells from both the treatment and vehicle control groups were then lysed using lysis buffer (kit content) for 45 min at 37 °C in a 5% CO_2_ incubator. The cell lysate (50 μL) was transferred to a new 96 well plate and 50 μL of assay buffer was added. The plate was incubated at room temperature for 30 min, and 50 μL of stop solution was added. The amount of formazan, which is proportional to the amount of LDH released from dead cells, was measured colorimetrically at 570 nm using a BioTek plate reader (BioTek Instruments, Winooski, VT, USA). The absorbance for background correction was determined to be 620 nm. The percentage of cell viability was calculated as follows: The % cell cytotoxicity = 100× (experimental well absorbance—negative control well absorbance)/(positive control well absorbance –negative control well absorbance). All calculations were performed after background absorbance correction and blank absorbance subtraction.

### 2.4. Quantitative Real-Time PCR

Total RNA was extracted from both TQ-treated and vehicle control samples using an RNA mini kit from (Qiagen, Manchester, UK), according to the manufacturer’s protocol. Total RNA was quantified using a NanodropTM (Thermo Fisher Scientific, Wilmington, MA, USA). cDNA was synthesized with 1 µg of RNA and random hexamers using ImProm-II reverse transcription kit according to the manufacturer’s protocol. Quantitative real-time PCR (qRT-PCR) was performed using a SYBR Green PCR kit (Qiagen, Manchester, UK). The sequences of the forward and reverse primers used for MCP-1, ICAM, and GAPDH (internal control) are listed in Table 1. PCR was performed in triplicate for each pair of primers using the StepOnePlus Real-time PCR machine (Applied Biosystems, Foster City, CA, USA). Results were normalized to the internal control, and gene quantitation was performed using the comparative ΔΔCt (ΔCt target − ΔCt control) method.

### 2.5. Monocyte Migration Assay

A cell migration assay was performed to study the migration behavior of THP-1 monocyte cells. Twenty-four-transwell inserts containing a membrane with pores of 8 μm (Corning, Corning, NY, USA) were used. Wells were split into separate halves to mimic the arterial endothelium layer while allowing monocyte migration. One million THP-1 monocytes in 200 μL of serum-free RPMI-1640 were loaded into the upper chamber of the Transwell insert. RPMI culture medium (600 µL) containing 20 ng/mL of MCP-1 (R&D Systems, Minneapolis, MN, USA) and TQ (5 and 10 µM) or TQ alone (5 and 10 µM) were added to the lower chamber. Control cells were treated with the vehicle alone (DMSO). The transwell chambers were then incubated at 37 °C in 5% (*v/v*) CO_2_ for 3 h to evaluate cell migration. Then, the medium in the upper chamber was removed and the cells present on the underside of the membrane were washed with PBS and collected in the lower chamber along with other migrated cells. The medium containing cells in the lower chamber was then collected and centrifuged (250× *g* for 5 min). The resultant cell pellet was resuspended in 2 mL of culture medium and counted using a hemocytometer. The number of THP-1 cells that migrated through the membrane to the lower chamber was calculated and expressed as a percentage of the number of cells that were originally added.

### 2.6. Cholesterol Efflux Assay

Cholesterol efflux was performed using a commercial cholesterol efflux assay kit (Cat No. ab196985) from (Abcam, Cambridge, UK), to investigate the effect of TQ on the progression of atherosclerosis in human THP-1 macrophages. Cells were seeded at a concentration of 1 × 105 cells in 100 μL of media per well in a 96-well black plate with a clear bottom. After differentiation into macrophages under PMA stimulation for 24 h, the human THP-1 macrophages were labeled with [3H] cholesterol carried by acetylated LDL for 24 h in RPMI 1640 supplemented with 0.2% BSA. After labeling, the cholesterol-loaded macrophages were washed with PBS, equilibrated with RPMI-1640, 0.2% BSA for 1 h, and then exposed to different concentrations of TQ (5 and 10 µM) for 24 h in a humidified 5% CO_2_ incubator at 37 °C. The positive control (20 μL) provided in the kit was added to 80 μL of RPMI representing the positive control wells, and 100 μL of serum-free RPMI was added to the negative control wells. For analysis of cholesterol efflux, the medium was collected and centrifuged at 6000× *g* for 10 min to remove cell debris and cholesterol crystals. The radioactivity content in an aliquot of the supernatant was determined by liquid scintillation counting (Ex/Em = 482/515 nm). The cells were lysed with 100 μL of cell lysis buffer and placed on a shaker for 30 min at RT. The percentage of cholesterol efflux was calculated by dividing the radioactivity intensity released from the cells into the medium relative to the sum of the radioactivity content in the cells and the medium.

$$\%Cholesterol\;Efflux=\frac{Intensity\;of\;Media}{Intensity\;of\;Media+Cell\;L\;ysate}\times 100$$

### 2.7. SwissTargetPrediction for TQ

The machine-readable format of the TQ structure was obtained, based on the canonical simplified molecular input line entry system (SMILES) from the PubChem Database [14,15,16]. The putative protein targets of TQ were obtained by virtual screening based on the “Similarity Principle” using SwissTargetPrediction [17,18,19]. In SwissTargetPrediction, putative binding predictions are accomplished from 376342 experimentally active analogous compounds in 2D and 3D that strongly interact with 3068 well-recognized protein targets [20,21], and the dataset is based on ChEMBL23. The putative protein targets are ranked in SwissTargetPrediction based on a score that merges both 2D and 3D similarity values of an active molecule to query molecules such as TQ [19]. A maximum of 100 putative protein targets were obtained as an output from the SwissTargetPrediction analysis [18,19] (Supplementary Figure S3 Flow Chart).

### 2.8. Web Gestalt Analysis of TQ Putative Protein Targets

To functionally classify the TQ-induced putative protein targets, the over-representation analysis (ORA) module of the wGSEA was used. In ORA, the preferred organism was *Homo sapiens*, and gene ontology (biological, cellular, and molecular functions) and disease association databases, such as OMIM, GLAD4U, and DisGeNET, were selected for further downstream analyses [16,22]. The default parameters for the enrichment analysis (minimum number of IDs (5), the maximum number of IDs (2000), the Benjamini–Hochberg (BH) method for computing the false discovery (FDR) rate (*p* < 0.05), and the significance level (Top 10) were chosen for each wGSEA analysis [16].

### 2.9. The Open Targets Platforms Analysis

The Open Targets Platform was utilized to uncover putative TQ protein targets associated with atherosclerosis [23,24,25]. Evidence from various omics studies, text mining of scientific publications, in-vivo models, and drugs are utilized in the Open Targets Platform to score and rank target-disease associations and assist target prioritization [24,25,26]. Here, the query lists with the putative molecular targets of TQ were used to decipher the diseases associated with atherosclerosis and other related disorders, such as carotid atherosclerosis, premature coronary artery atherosclerosis, coronary atherosclerosis, and cerebral atherosclerosis (Supplementary Table S1).

### 2.10. Ingenuity Pathway Analysis

Ingenuity Pathway Analysis (IPA) software has a cutting-edge next-generation knowledge base that consists of clarified scientific information from publications, databases, and other relevant resources [16,27]. Here, we applied the IPA software (Qiagen, Manchester, UK) to functionally annotate the protein clusters and identify biologically significant disease-specific pathways regulated by TQ molecular targets. The putative molecular targets of TQ were subjected to core analysis in the IPA to delineate biologically relevant molecular networks related to atherosclerosis, using the right-tailed Fisher’s exact test and Benjamini–Hochberg correction (BHC) for multiple testing (*p* < 0.05). In addition, we used the molecular activity prediction algorithm in IPA to predict the regulation of putative molecular targets of TQ in atherosclerosis.

### 2.11. Statistical Analysis

Other than independent statistical details mentioned in the above sections, generally, all data are expressed as the mean ± SEM from at least three independent experiments (unless specified) as indicated in figure legends. Statistical comparisons between different groups were assessed by one-way ANOVA followed by Student–Newman–Keuls post hoc test with GraphPad Prism software (version 8.0). A value of *p* < 0.05, was considered to indicate a statistically significant intergroup difference [28]. In addition, cytoscape, FunCoup, and KEGG pathway enrichment analysis was performed [29,30,31].

## 3. Results

### 3.1. TQ Did Not Affect the Viability of THP-1 Macrophages

**Figure 1:**
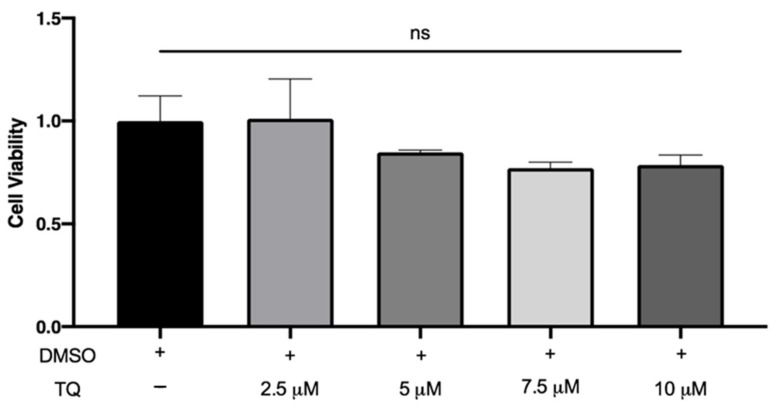
The effect of TQ on human macrophage viability. PMA differentiated human THP-1 macrophages were incubated for 24 h in RPMI medium 1640 containing the indicated concentrations of Thymoquinone (TQ). Cell viability was evaluated using lactate dehydrogenase assay and data were normalized to cells treated with DMSO as a vehicle control arbitrarily assigned as 1.0. The data were presented as the mean ± SEM from three independent experiments. ns: non-significant.

LDH is a cytoplasmic enzyme released in the extracellular medium when the plasma membrane is damaged. There was no significant increase in LDH release by macrophages following incubation with 2.5, 5, 7.5, and 10 µM TQ compared to vehicle-treated cells, indicating that TQ was not cytotoxic (Figure 1).

### 3.2. TQ Inhibits IFN-γ-Induced ICAM-1 and MCP-1 Gene Expression in Human THP-1 Macrophages

**Figure 2:**
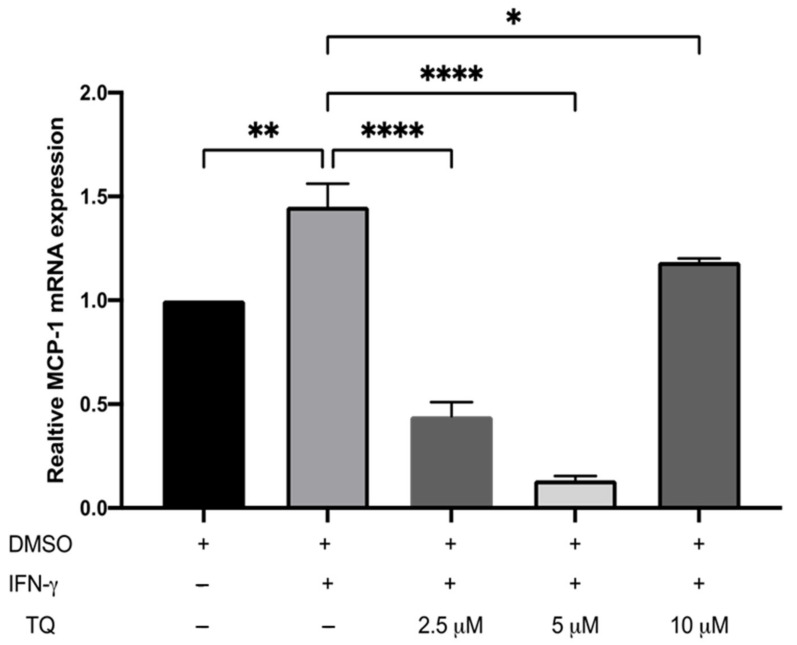
The effect of TQ on the IFN-γ induced MCP-1 expression in human THP-1 macrophages. Gene transcript level of MCP-1 was assessed in PMA differentiated human THP-1 macrophages treated with IFN-γ (250 U/mL) or IFN-γ and the indicated concentrations of TQ for 3 h. Data were normalized to cells treated with DMSO as vehicle control. Total RNA was subjected to RT-qPCR with primers specific for human MCP-1 or GAPDH. Gene transcript levels were calculated using the comparative Ct method and normalized to the GAPDH level of vehicle-treated cells. The data are presented as the mean ± SEM from three independent experiments. Statistical analysis was performed using one-way ANOVA. ‘*’ indicates statistical significance where *: *p* < 0.05; **: *p* < 0.01; ****: *p* < 0.0001.

**Figure 3:**
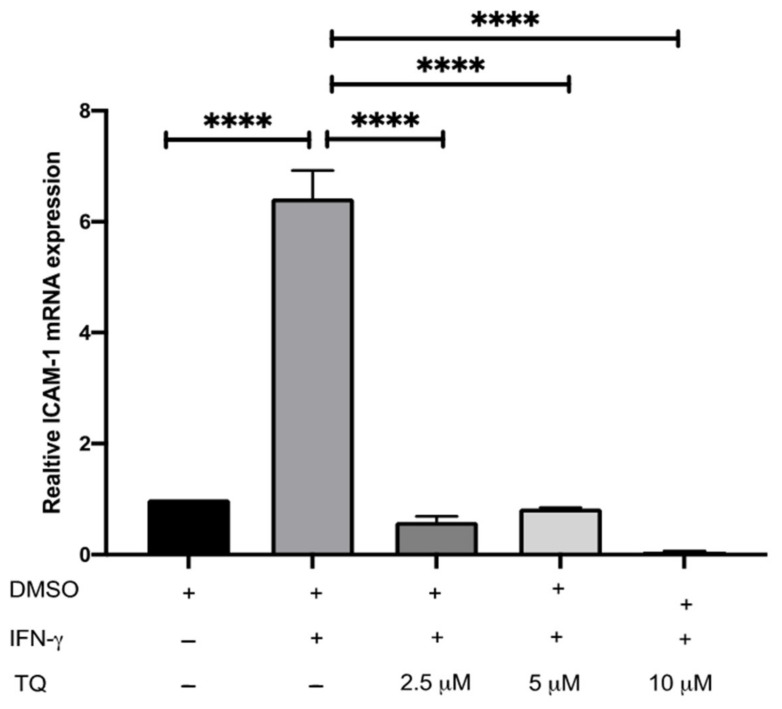
The effect of TQ on the IFN-γ induced ICAM-1 expression in human THP-1 macrophages. Gene transcript level of ICAM-1 was assessed in PMA differentiated THP-1 macrophages treated with IFN-γ (250 U/mL) or IFN-γ (250 U/mL) and the indicated concentrations of TQ for 3 h. Data were normalized to cells treated with DMSO as vehicle control. Total RNA was subjected to RT-qPCR with primers specific for human ICAM-1 or GAPDH. Gene transcript levels were calculated using the comparative Ct method and normalized to the GAPDH level of vehicle-treated cells. The data are presented as the mean ± SEM from three independent experiments. Statistical analysis was performed using one-way ANOVA. ‘*’ indicates statistical significances where ****: *p* < 0.0001.

The expression of ICAM-1 and MCP-1 in THP-1 macrophage cells following treatment with either vehicle (control), IFN-γ, or IFN-γ with TQ for 24 h was evaluated using qRT-PCR. THP-1 macrophages stimulated with IFN-γ (250 U/mL) showed a significant increase in MCP-1 and ICAM-1 expression (1.45- and 6.48- fold, respectively) compared to the control (Figure 2 and Figure 3). Treatment with TQ (2.5 and 5 µM) induced a decrease in the expression of MCP-1 compared to the control (0.39- and 0.63-fold, respectively) in the presence of IFN-γ. In contrast, treatment with 10 µM TQ induced an increase in MCP-1 expression compared to that in the control (Figure 2). The expression of ICAM-1 was significantly decreased in response to treatment with TQ (2.5, 5, and 10 µM) compared to the control (0.6-, 0.31-, and 0.87-fold, respectively) in the presence of IFN-γ (Figure 3). Compared to IFN-γ induction, the expression of both ICAM-1 and MCP-1 following treatment with TQ (2.5, 5, and 10 µM) decreased at all concentrations. The decreases in the expression of MCP-1 (by 57.8%, 74.61%, and 17.9%) and ICAM-1 (by 93.7%, 89.4%, and 97.9%, respectively) were statistically significant compared to IFN-γ induction alone Figure 2 and Figure 3.

### 3.3. TQ Inhibits Human Monocyte Migration towards MCP-1

**Figure 4:**
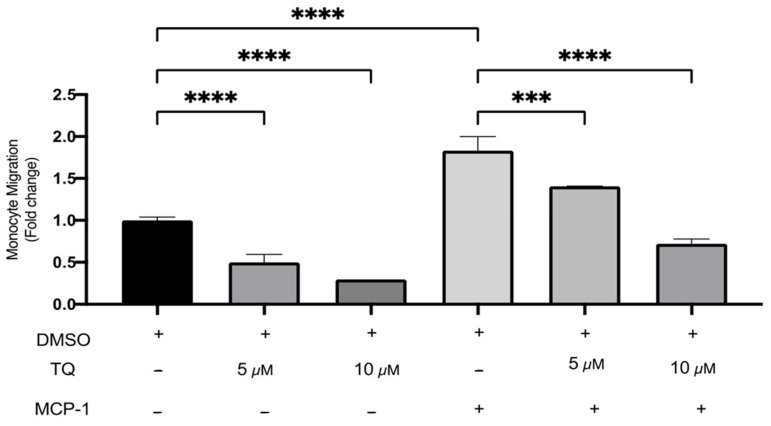
The effect of TQ on the migration of human THP-1 monocytes. The effects of TQ on THP-1 monocytes’ cellular migration were assessed with or without MCP-1 (20 ng/mL) in the presence of 5 or 10 µM of TQ for 3 h. Data were normalized to cells treated with DMSO as vehicle control. Monocyte migration is expressed as fold-change compared to the proportion of cells that moved from the upper chamber to the lower chamber in vehicle-treated cells. The data are presented as the mean ± SEM from two independent experiments. Statistical analysis was performed using one-way ANOVA. ‘*’ indicates the statistical significance of *** *p* < 0.001 and **** *p* < 0.0001.

The effects of TQ on monocyte migration were evaluated using a transwell chamber with or without MCP-1 (20 ng/mL) stimulation. TQ effectively decreased monocyte migration relative to vehicle control. There was a dose-dependent decrease of 50.0% and 70.4% with 5 µM (*p* < 0.0001) and 10 µM (*p* < 0.0001) of TQ Figure 4. As expected, MCP-1, which is a major chemoattractant for monocytes, effectively increased migration to the lower transwell chamber compared to unstimulated controls. There was a significant increase of 83.3% (*p* < 0.0001) (Figure 4). In the presence of MCP-1, TQ treatment (5 and 10 µM) induced a significant dose-dependent decrease in THP-1 monocyte migration. The decrease in migration was 23.2% (*p* < 0.001) and 60.6% (*p* < 0.0001), respectively (Figure 4). Collectively, the data show that TQ directly inhibits monocyte migration, even in the presence of MCP-1 induction.

### 3.4. TQ Has no Effect on Cholesterol Efflux from Human THP-1 Macrophages

**Figure 5:**
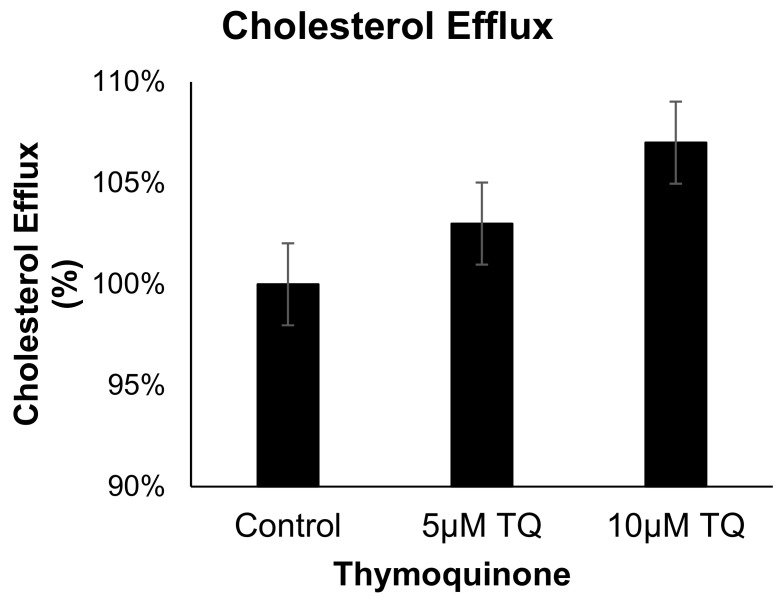
The effect of TQ on cholesterol efflux in THP-1 macrophages. Cholesterol efflux was assessed using cholesterol-loaded THP-1 cells treated with 5 or 10 µM of TQ for 24 h. Cholesterol efflux was calculated as a percent of media [3H]cholesterol per total cell and media [3H]cholesterol. Data were normalized to cells treated with DMSO as vehicle control. The data are presented as the mean ± SEM from two independent experiments.

The effect of TQ (5 and 10 µM) on cholesterol efflux in cholesterol-loaded THP-1 macrophages was not significantly different from that of the control Figure 5. These results indicate that the anti-atherosclerotic effect of TQ is not mediated by cholesterol efflux acceleration in THP-1 macrophages.

### 3.5. In-Silico Analysis Reveals TQ Molecular Targets Implicated in Atherosclerosis

**Figure 6:**
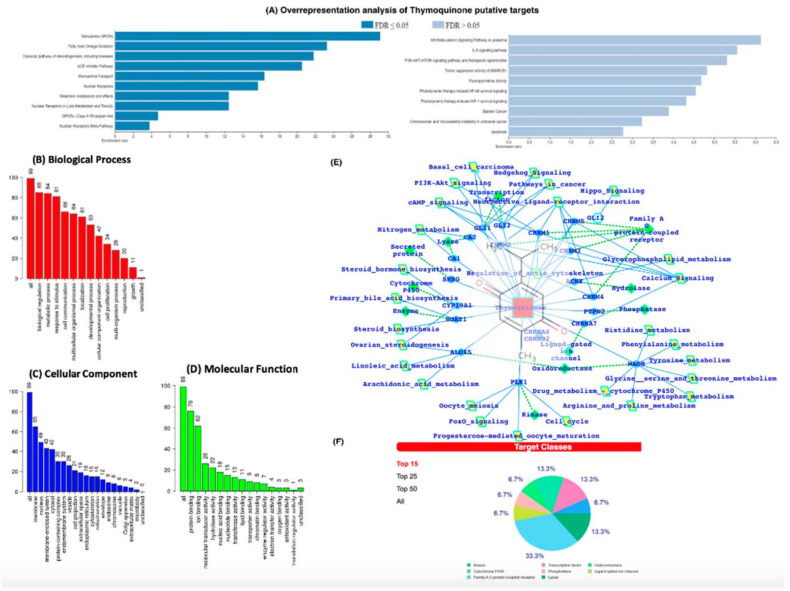
(**A**) Over Representation Analysis of putative targets of TQ using the Wiki pathway database and Wiki pathway Cancer database. (**B**–**D**) GO (Gene Ontology) terms enrichment analysis. (**E**) TQ target proteins predicted by SwissTargetPrediction. TQ targets have been presented with the respective protein classes and the associated pathways. (**F**) Pie chart representing the TQ targets percent distribution for the protein classes.

We then conducted an in-silico analysis to predict the putative molecular targets of TQ. To this end, we used the SwissTargetPrediction tool with canonical SMILES computed by OEChem (Version 2.1.5). According to a previous study, the highest probability of binding of TQ was with serine-threonine protein kinase 13 (STPK13) or Polo-like Kinase-1 (PLK-1) (ref PMID: 23135290, Figure 6B–D). In addition, we also identified the probability of binding to 19 other proteins, including GLI2, GLI1, ALOX5, CYP19A1, MAOB, PTPN2, CHRNA4, CHRNB2, ACHE, CA2, CA1, CHRM4, CHRM5, CHRM2, CHRM1, CHRM3, SOAT1, SHBG, CHRNA7, and ACE (Supplementary Table S1). The putative protein target list contained 100 user IDs for TQ, in which 99 user IDs were unambiguously mapped to 99 unique Entrez gene IDs and one user ID was not mapped to any Entrez gene ID. Among the 99 unique Entrez gene IDs, nine IDs were annotated to the selected functional categories (Supplementary Table S1). The reference lists consist of all mapped Entrez gene IDs from the selected platform genome. GO Slim Summary for TQ putative molecular targets in humans displayed biological process, cellular component, and molecular function category in the red, blue, and green bars, respectively. The height of the bar characterizes the number of IDs in the user list, as well as in the category (Figure 6B–D). Moreover, we have also analyzed the TQ partner proteins (Figure 6E) by using the SwissTargetPrediction (http://www.swisstargetprediction.ch/) (accessed on 1 July 2021) web server which further predicts the protein classes (Figure 6F). In Figure 6E,F, it is clear that GPCRs, Lyases, TFs, and Kinases are dominantly present and a number the proteins which are the potential targets of TQ are known to be associated with the immune and inflammatory systems.

The ORA using the Wiki pathway and Wiki pathway Cancer pathway databases showed that the putative protein targets of TQ significantly regulate several pathways, including fatty acid oxidation, ACE inhibitor pathway (Figure 6A), IL-6 signaling pathway, and NF-kB signaling pathway (Figure 6A).

Next, we used the Open Targets Platform to determine the association between diseases and the putative molecular targets of TQ. Our findings showed that among the list of 99 putative targets of TQ, approximately 60 targets were associated with atherosclerosis, 12 with carotid atherosclerosis, six with premature coronary artery atherosclerosis, six with coronary atherosclerosis, and four with cerebral atherosclerosis (Supplementary Table S1). In addition, 32 putative targets of TQ were associated with hypercholesterolemia (Supplementary Table S2) and 24 with hypertriglyceridemia (Supplementary Table S3). The IPA core analysis of the molecular targets of TQ involved in atherosclerosis revealed that 27 molecules (ACE, CA1, CA12, CA13, CA14, CA2, CA5B, CA6, CA7, CA9, CNR1, CYP2C19, IL6, IMPDH2, MTNR1A, MTNR1B, NOS3, NR3C1, NR3C2, PDE7A, PGR, PPARA, PPARG, PTGS2, SOAT1, TLR9, XIAP) were involved in the regulation of atherosclerosis (Supplementary Figure S1). The molecular activity prediction analysis showed that TQ inhibition of IL-6 significantly attenuated the levels of Akt, NF-kB Complex, Ap1, ERK ½ STAT5 a/b, PTGS2, PI3K Complex, XIAP, and estrogen receptor in atherosclerosis (Supplementary Figure S2).

## 4. Discussion

Atherosclerosis is a major healthcare and economic burden, and many avenues are pursued to prevent, limit, or treat it. Conventionally, atherosclerosis is treated using anti-cholesterol agents (statins/fibrates), antiplatelet agents (aspirin), and beta-blockers. Despite the well-established lipid-lowering effects of statins and their benefits in reducing the clinical complications of atherosclerosis, many clinical trials have highlighted substantial residual cardiovascular risk [32,33,34]. In addition, the outcomes of many agents against established targets (e.g., inhibitors of cholesterol ester transfer protein, acyl-CoA acyltransferase-1, and lipoprotein-associated phospholipase A2, agonists of peroxisome proliferator-activated receptor) have remarkable limitations [8,35]. Therefore, it is essential to identify new synthetic or natural therapeutic agents that might effectively control atherosclerosis and scientifically validate them.

Several natural extracts exhibit anti-atherosclerotic properties. *Ziziphus nummularia* extract suppressed TNF-α-induced adhesion of THP-1 monocytes to human aortic smooth muscle cells (HASMCs) and endothelial cells in a concentration-dependent manner [36]. β-Elemene isolated from *Curcuma wenyujin* reduced the size of atherosclerotic lesions and increased plaque stability in apo-E-knockout mice by inhibiting the production of pro-inflammatory cytokines and cell adhesion molecules, such as IL-1β, TNF-α, IFN-γ, MCP-1, and ICAM-1 [37]. The ethanol extract of *Prunella vulgaris* suppressed the adhesion of monocyte-/macrophage-like human macrophage cells (THP-1 cells). *P. vulgaris* also decreased the expression of ICAM-1 and VCAM-1, the amount of reactive oxygen species (ROS), E-selectin, and NO production in TNF-α-induced HASMCs [38]. Danshenol A from *Salvia miltiorrhiza* suppressed ICAM-1 expression induced by tumor necrosis factor-α (TNF-α) and monocyte adhesion to endothelial cells [39]. Punicalagin inhibits the production of pro-inflammatory cytokines and the expression of MCP-1 and ICAM-1 in human differentiated macrophage THP-1 cells [40].

TQ is the main active compound in *Nigella sativa* seeds and is widely known and used in herbal medicine. Many reports have shown that TQ has potent anti-inflammatory and antioxidant activities both in vitro and in-vivo. Treatment of a pancreatic cancer cell line (HS766T) with TQ (25–75 μM) for 6 h led to the complete abolishment of MCP-1 expression [41]. In addition, treatment of synovial fibroblasts derived from rheumatoid arthritis patients with TQ (1–5 μM) for 24 h decreased ICAM-1 gene expression despite pre-stimulation with TNF-α [42]. In addition, TQ treatment inhibits the pro-inflammatory response of hepatic stellate cells exposed to lipopolysaccharide and confers protection to the liver [43]. However, few studies have focused on their potential anti-atherogenic actions. This study investigated the effect of TQ on inflammation and monocyte recruitment in human THP-1 macrophages and established that TQ significantly inhibited ICAM-1 and MCP-1 gene expression in response to IFNγ. In addition, we conducted in-silico analysis of TQ, which revealed new putative targets involved in pathways associated with atherosclerosis.

The anti-inflammatory actions of TQ could be linked to the suppression of the nuclear factor kappa-light-chain-enhancer of activated B cells (NF-κB). This protein complex has been mainly considered a proinflammatory pathway due to its activation of proinflammatory cytokines, such as the interleukin-1 family. Indeed, TQ administration has been shown to reduce NF-κB expression in diabetic rats and hepatic stellate cells [43]. To understand the mechanism of action of TQ, it would be useful to investigate these protein complexes in human THP-1 macrophages. In addition, our current study mainly focused on the effect of TQ at the cellular level, but the effects of TQ on atherosclerosis require further studies in animal models.

In this study, we found that TQ reduced the migration of THP-1 monocytes both in the absence or presence of MCP-1, indicating that TQ could be effective even in the absence of inflammation. In line with our results, previous in vitro studies have also demonstrated that TQ reduces the migration of many different cell types, including vascular smooth muscle cells (VSMCs), human alveolar basal epithelial adenocarcinoma cells (A549), and human glioblastoma cells (U-87 and CCF-STTG1) [44,45,46]. Therefore, it is clear that TQ exerts anti-migratory properties, which could be beneficial in many clinical conditions.

Many studies have reported that TQ has hypolipidemic effects. Oral administration of TQ (3.5 mg/kg/day) in rabbits for four weeks led to a reduction in the levels of total cholesterol [10]. Similarly, the atherogenic diet-fed rats treated with TQ (10 mg/kg) for 30 days led to a significant reduction in the total cholesterol ratio compared to the control group [47]. Intraperitoneal injection of TQ 0.5–8 mg/kg/day for two weeks in rats resulted in the reduction of blood cholesterol [48]. Another in vivo study on NZW rabbits fed a cholesterol diet together with N. sativa extract for eight weeks demonstrated a significant decrease in low-density lipoprotein (LDL) and total cholesterol (TC) compared to the untreated group fed with cholesterol alone [11]. In this study, the cholesterol efflux assay revealed that TQ does not affect the reverse cholesterol transport of monocytes, indicating that the anti-hypercholesterolemic properties of TQ involved other cell types. All of the above studies confirmed that TQ can be beneficial against hypercholesterolemia.

A previous study reported that the administration of propolis (another component of *Nigella sativa* seed oil) or TQ both inhibited the formation of early atherosclerotic lesions in hypercholesterolemic rabbits through a decrease in total cholesterol (TC), LDL-C, and triglyceride (TG) associated with higher HDL-C levels [10]. The authors associated these protective effects with a reduction in oxidative damage resulting from a high-cholesterol diet as ethanolic propolis extract or TQ treatment reduced serum thiobarbituric acid reactive substances (TBARS) levels and enhanced glutathione levels in rabbits fed a high-cholesterol diet [10]. Therefore, it would be interesting to test whether the reduction of MCP-1 and ICAM-1 expression and monocyte migration revealed in this study could depend on the antioxidant properties of TQ.

## 5. Conclusions

This study investigated the mechanism of action of the TQ. We showed that stimulated ICAM-1 and MCP-1 gene expression in response to IFN-γ was significantly decreased by TQ treatment. We also showed that TQ decreased monocyte migration and had no effect on cholesterol efflux in human THP-1 macrophages. Taken together, these findings show that TQ has anti-inflammatory effects and can be considered as a nutraceutical molecule for the prevention and treatment of atherosclerosis.

## Figures and Tables

**Table 1:** Genes and primer sequences.

Genes	Primer Sequence
MCP-1	F: 5′-CGCTCAGCCAGATGC-AATCAATG-3′ R: 5′-ATGGTCTTGAAGATCA-CAGCTT-CTTTGG-3′
ICAM-1	F: 5′-GACCAGAGGTTGAAC-CCCAC-3′ R: 5′-GCGCCGGAAAGCTG-TAGAT-3′
GAPDH	F: 5′-CTTTTGCGTCGCCAG-CCGAG-3′ R: 5′-GCCCAATACGACCAAA TCCGTTGACT-3′

## Data Availability

Not applicable.

## Supplementary Materials

The following supporting information can be downloaded at https://www.mdpi.com/article/10.3390/cimb44040120/s1, Figure S1: Ingenuity pathway analysis of putative targets of TQ. Core analysis of the targets of TQ involved in atherosclerosis. Figure S2: Molecular activity prediction of the effect of TQ on the IL -6 signaling pathway. The schematic shows activation of the IL6 pathway in the absence (A) or presence of TQ (B). The orange color indicates predicted activation, the blue color indicates predicted downregulation, the grey color indicates unpredicted effects, the green color indicates decreased measurement, and the red color indicates increased measurement. Figure S3: Flowchart illustrating the important steps of in silico analysis using next-generation knowledge discovery (NGKD) platforms to unravel the molecular targets of thymoquinone and its link to atherosclerosis. Table S1: List of putative protein targets of TQ. The probability score was determined using SwissTarget Prediction. Table S2: Disease association of putative targets of TQ. The *p*-value was determined using the Open Targets Platform. Table S3: Putative targets of TQ associated with hypercholesterolemia. The *p*-value was determined using the Open Targets Platform.
