# Supplementary figures and images for: Thymoquinone (TQ) Inhibits Inflammation and Migration of THP-1 Macrophages: Mechanistic Insights into the Prevention of Atherosclerosis Using In-Vitro and In-Silico Analysis
Source: Curr Issues Mol Biol. 2022 Apr 15;44(4):120. doi: 10.3390/cimb44040120 (PMC9164073; doi:10.3390/cimb44040120)

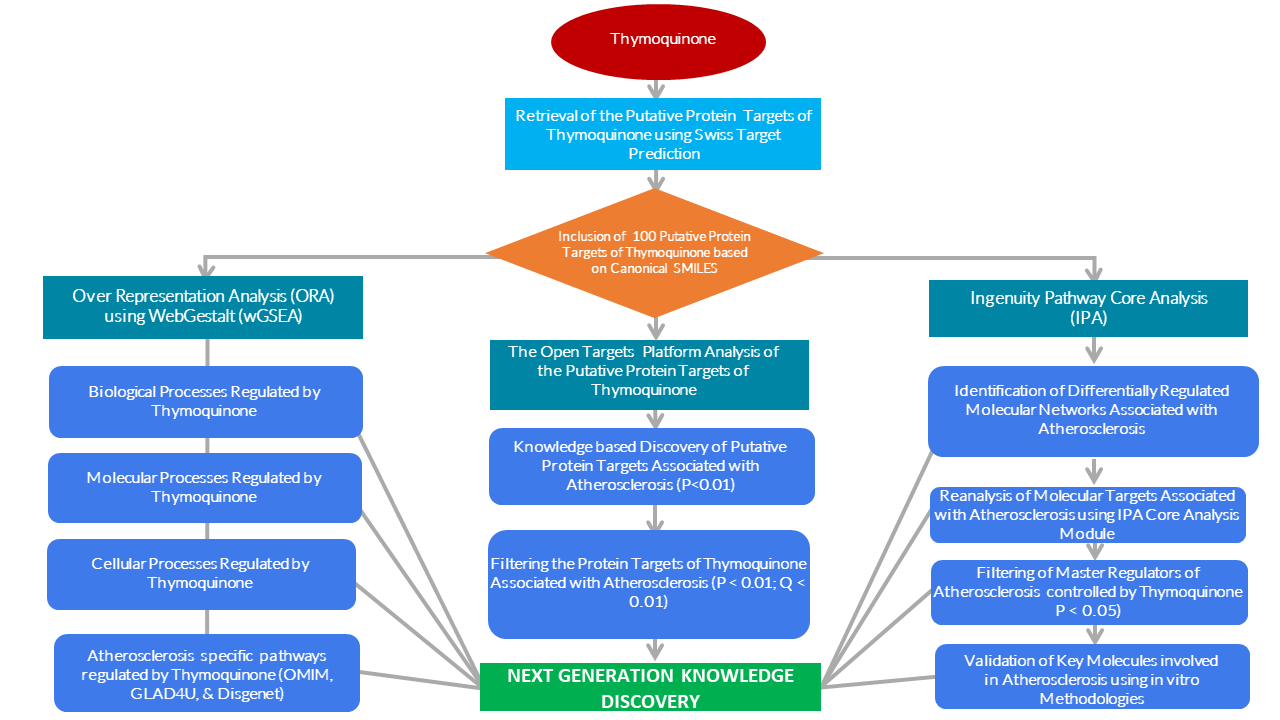

Supplement: Supplementary file 1 [file cimb-44-00120-s001.zip › Figure S3.tif]

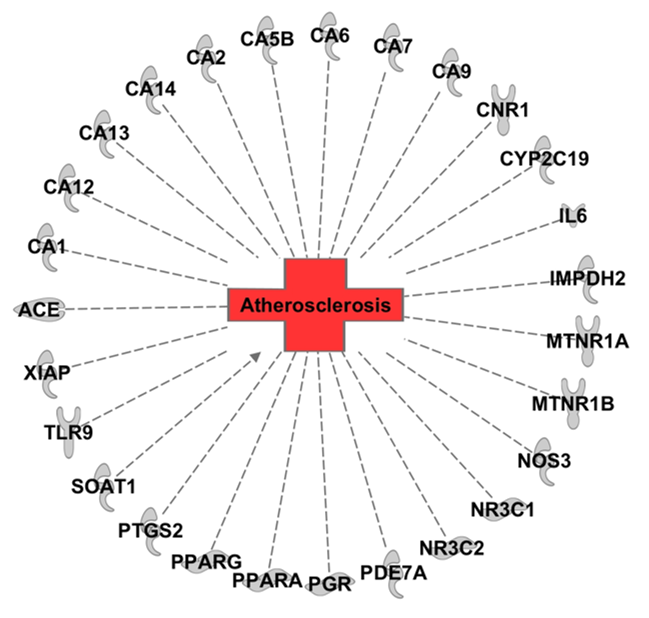

Supplement: Supplementary file 1 [file cimb-44-00120-s001.zip › Figure S1.tif]

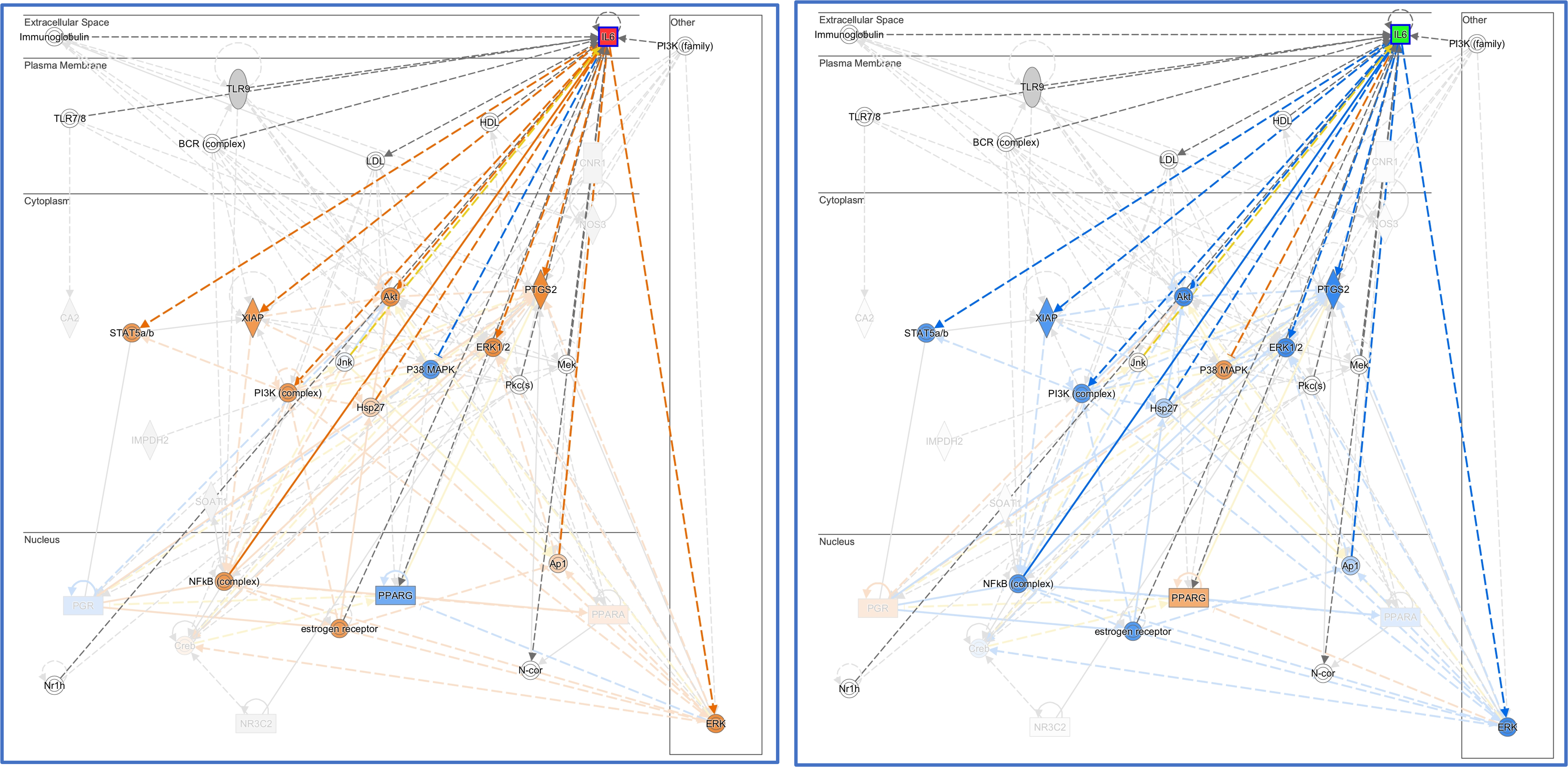

Supplement: Supplementary file 1 [file cimb-44-00120-s001.zip › Figure S2.tif]
